# Improvement of Skeletal Muscle Regeneration by Platelet-Rich Plasma in Rats with Experimental Chronic Hyperglycemia

**DOI:** 10.1155/2020/6980607

**Published:** 2020-07-11

**Authors:** Raed Rtail, Olena Maksymova, Viacheslav Illiashenko, Olena Gortynska, Oleksii Korenkov, Pavlo Moskalenko, Mohamad Nasser, Gennadii Tkach

**Affiliations:** ^1^Department of Morphology, Sumy State University, Sumy, Ukraine; ^2^Department of Family Medicine with Dermatovenerology, Sumy State University, Sumy, Ukraine; ^3^Department of Dentistry, Sumy State University, Sumy, Ukraine; ^4^Lebanese University, Hadath, Lebanon

## Abstract

Herein, the structural effect of autologous platelet-rich plasma (PRP) on posttraumatic skeletal muscle regeneration in rats with chronic hyperglycemia (CH) was tested. 130 white laboratory male rats divided into four groups (I—control; II—rats with CH; III—rats with CH and PRP treatment; and IV—rats for CH confirmation) were used for the experiment. CH was simulated by streptozotocin and nicotinic acid administration. Triceps surae muscle injury was reproduced by transverse linear incision. Autologous PRP was used in order to correct the possible negative CH effect on skeletal muscle recovery. On the 28th day after the injury, the regenerating muscle fiber and blood vessel number in the CH+PRP group were higher than those in the CH rats. However, the connective tissue area in the CH group was larger than that in the CH+PRP animals. The amount of agranulocytes in the regenerating muscle of the CH rats was lower compared to that of the CH+PRP group. The histological analysis of skeletal muscle recovery in CH+PRP animals revealed more intensive neoangiogenesis compared to that in the CH group. Herewith, the massive connective tissue development and inflammation signs were observed within the skeletal muscle of CH rats. Obtained results suggest that streptozotocin-induced CH has a negative effect on posttraumatic skeletal muscle regeneration, contributing to massive connective tissue development. The autologous PRP injection promotes muscle recovery process in rats with CH, shifting it away from fibrosis toward the complete muscular organ repair.

## 1. Introduction

Skeletal muscle injuries account for about 30% of all occupational diseases in industrialized countries [1]. Muscle trauma is also one of the main reasons for the decline of athlete competition performance [2]. Therefore, uncovering the cellular and molecular mechanisms of skeletal muscle regeneration and the development of effective ways of muscle recovery improvement are the important tasks of modern medical science [3].

Currently, chronic hyperglycemia (CH) is one of the most common metabolic disorders worldwide [4]. CH is associated with the development of secondary complications in skeletal muscle and may impair its regeneration capacity. Experimental studies have shown that CH attenuates the expression of muscle-specific transcription factors (MyoD and myogenin) [5] and reduces the number of myosatellite cells (MSCs) in both animal and human skeletal muscle [6, 7]. Jeong et al. have shown that MSCs derived from rats with streptozotocin-induced diabetes mellitus (DM) are incapable of myotube formation [8]. Also, rats with genetic DM models have a significant delay and incompleteness of posttraumatic skeletal muscle regeneration [9, 10].

Platelet-rich plasma (PRP) is one of the promising therapeutic agents capable of enhancing regeneration of various tissues and organs, including striated muscles. Wright-Carpenter et al. [11] revealed that autologous conditioned serum administration promotes MSC activation and increases the size of the regenerating fibers within injured skeletal muscle. Gigante et al. [12] had found that platelet-rich fibrin matrix improves regeneration and promotes neovascularization in striated muscle after mechanical lesion. Several studies have also shown that PRP administration reduces the regeneration time and improves histologic outcome and functional recovery of the skeletal muscle [13–16].

Hamid et al. [17] showed that PRP is able to accelerate the recovery of injured skeletal muscle in athletes. Nitecka-Buchta et al. [18] have reported that PRP injection into the masseter muscle reduces pain in patients with temporomandibular disorders. However, a few systematic reviews have failed to conclude on PRP effectiveness and safety for human muscular injury treatments [19–21].

PRP does have a positive effect on soft tissue repair in patients with DM. The results of meta-analysis have shown that PRP significantly improves wound healing and accelerates functional recovery in patients with diabetic foot syndrome [22–24]. Regrettably, there is no current work devoted to investigation of the PRP effect on skeletal muscle regeneration under CH influence.

The aim of the study was to investigate the structural effects of autologous PRP on posttraumatic skeletal muscle regeneration in rats with CH.

## 2. Materials and Methods

### 2.1. Animals

130 white laboratory male rats (age—7-9 months) were used for the experiment. All the animals were divided into four groups: I—control group (40 rats with skeletal muscle injury); II—CH group (40 animals with simulated CH and skeletal muscle injury); III—CH+PRP group (40 rats with simulated CH and skeletal muscle injury, which received PRP injection into muscle damage area); and IV—CH confirmation group (10 animals with CH for glucose homeostasis evaluation).

All the animals, participating in the test, were examined for motor activity and outer covering condition. Then, rats were subjected to a two-week quarantine. The experimental animals were used according to policies of general ethical principles of experiments on animals (Kyiv 2001), Declaration of Helsinki (2000), European Convention for the Protection of Vertebrate Animals used for Experimental and Other Scientific Purposes (Strasbourg 1985). Ethics and morality were not violated during the research. Rats were housed in the vivarium room under constant temperature (24-25°C), humidity (60 ± 5) %, and 12-hour dark-light cycle. Cage cleaning was performed daily.

### 2.2. Simulation of Chronic Hyperglycemia and Muscle Trauma

The rats of II, III, and IV groups have been consuming 10% aqueous fructose solution instead of drinking water for 2 weeks. Then, a single intraperitoneal injection of streptozotocin (40 mg/kg, Sigma-Aldrich, USA), dissolved in citrate buffer (pH 4.5), and nicotinic acid (1 mg/kg) for each animal was performed. Each control rat received a single intraperitoneal citrate buffer (pH 4.5) injection. Following streptozotocin or vehicle administration, animals were placed under normal vivarium conditions with a normal diet (food and water ad libitum) for 60 days.

Animals of the IV group were used to assess glucose homeostasis and to confirm the CH. Fasting glucose level, insulin, and C-peptide concentration were determined in the blood of these rats on the 60th day after CH simulation. Obtained data were used to confirm the CH presence.

### 2.3. Skeletal Muscle Injury

The injury model was modified from a rat model described by Tsai et al. [16]. The trauma of triceps surae muscle was reproduced in rats of I, II, and III groups 60 days after CH simulation. Surgery was performed in aseptic conditions under ketamine (8 mg/kg) and xylazine (3 mg/kg) anesthesia. The mechanical injury was applied by transverse linear incision of a lateral head of the triceps surae muscle at a point that is 40% distal to its origin. Ophthalmic knife (blade width—1.8 mm; blade length—4.5 mm) was used for traumatization. The defect width was approximately 75% of the muscle width; the defect depth was approximately 50% of the muscle thickness. At the end of the operation, the wound edges were matched and the skin was sutured.

### 2.4. PRP Preparation

A recent study showed that PRP promotes muscle regeneration and decreases inflammation and apoptosis in the injury model described above [16]. This allowed us to move forward and evaluate the effects of PRP on muscle recovery in rats with this injury model in the context of CH. In order to correct a possible negative CH effect on skeletal muscle regeneration, the autologous PRP (250 *μ*l) was injected into the wound of III group animals before the suturing. Previously, 0.8 ml of blood from the lateral tail vein was collected into vacutainers containing sodium citrate solution. The lost blood volume was immediately restored by sterile saline infusion. The selected blood was centrifuged (20 min; speed—2,000 rpm). As a result, two blood component fractions were observed in the test tube: lower dark red fraction (cellular components) and an upper straw yellow fraction (serum components). The upper fraction and upper portion of the lower fraction were pipetted and transferred to another tube. The resulting material was centrifuged (15 min; speed—2,000 rpm), which led to the formation of two fractions: lower, platelet-rich plasma, and upper, platelet-poor plasma. The lower fraction was transferred to a sterile tube, and the volume was adjusted to 0.5 ml with 10% calcium chloride solution [25]. The resulting solution was injected into the muscle wounds of III group animals. Each animal received its own (autologous) plasma.

### 2.5. Histology and Morphometry

The morphological features of skeletal muscle regeneration in I, II, and III groups were studied on the 3rd, 7th, 14th, and 28th day after receiving the mechanical injury. Animals were removed from the experiment (10 rats per term) by thiopental anesthesia overdose (4 mg/100 g body weight).

The portions of injured skeletal muscles were fixed in a 10% formalin solution in order to study the microscopic structure. The samples were dehydrated in alcohols of increasing concentration and then placed into paraffin. Transverse sections (across the muscle fibers) were made using MC-2 microtome (thickness—4-6 *μ*m). The staining was performed with hematoxylin-eosin (to evaluate the number and cross-sectional area of different muscle fibers, to determine vessel number, and to assess the inflammatory infiltration) and picrosirius red (to evaluate the collagen content and its formation dynamics). Semithin sections (thickness—1 *μ*m) stained with methylene blue were also made to visualize and count different white blood cells. The lateral head of triceps surae muscle sampling in each group was performed on all 10 rats.

An Olympus BH-2 microscope (Japan) was used for light microscopy. Images of histological specimens were performed using a Baumer/Optronic Typ: CX 05c digital camera. The morphometric analysis was done using microgrid, microwave line, and Digimizer computing software (version 5.3.5). All muscle fibers were divided into three types: normal muscle fibers (NMF)—typical muscle fibers without injury signs; damaged muscle fibers (DMF)—fibers with atypical shape and size along with signs of damage; and regenerating muscle fibers (RMF)—centrally nucleated fibers. Two-dimensional analysis of cross-sectional samples was carried out to determine the number of DMF (no./mm^2^), RMF (no./mm^2^), vessels (no./field), the regeneration area, the connective tissue area, granulocyte (no./mm^2^), and agranulocyte number (no./mm^2^). The regeneration area was calculated as the percentage of regenerating fibers from the total muscle fiber area. Connective tissue area was defined as the percentage of connective tissue area of the total muscle cross-sectional area. The qualitative summary assessment of the following histopathological changes was also carried out: necrosis (muscle fibers with impaired membrane integrity, vacuolization, and sarcoplasm disorganization); connective tissue edema (increasing of connective tissue spaces without signs of new fiber formation); inflammatory infiltration (amount of white blood cells); vascularization (number of vessels); fibrosis (area of new collagen fibers); and a number of centrally nucleated muscle fibers.

### 2.6. Statistical Analysis

Mathematical analysis was performed using SPSS (version 17.0, USA). Continuous data are presented as mean (M) ± standard deviation (SD). Kolmogorov-Smirnov test was used to check the normality distribution. The significance of differences between groups was determined using Student's *t*-criterion and one-way ANOVA followed by Bonferroni post hoc test. *P* values < 0.05 were considered statistically significant.

## 3. Results

The results of blood biochemical analysis of control (group I) and experimental animals (group IV) on the 60th day after CH simulation are shown in Table 1. Rats with streptozotocin-induced CH had significantly higher fasting glucose level (*P* < 0.001) and decreased insulin content (*P* = 0.005). The C-peptide amount did not differ between comparison groups (*P* = 0.267). CH rats also had higher concentration of total cholesterol (*P* < 0.001), triglycerides (*P* < 0.001), and LDL (*P* < 0.001) and decreased HDL level (*P* = 0.004). Obtained changes largely correspond to the type 2 DM phenotype.

The results of estimating the muscle fibers and vessel amount in skeletal muscle of different groups on 3rd, 7th, 14th, and 28th days after mechanical injury are presented in Table 2. The significant difference in the mean values of all studied parameters between rats of I, II, and III groups was revealed at each experiment time (according to ANOVA). The results of post hoc test on the 28th day after injury showed that DMF number in control rats was lower compared to that in CH (by 68.4%; *P* < 0.001) and CH+PRP group (by 53.8%; *P* < 0.001). DMF number in animals with CH was higher than that in CH+PRP rats (by 31.6%; *P* < 0.001). In contrast, the RMF and blood vessel number on the 28th day after injury in control animals was higher compared to that in CH rats (by 26.8%; *P* < 0.001—for RMF; by 40%; *P* < 0.001—for vessels) and CH+PRP rats (by 7.9%; *P* = 0.030—for RMF; by 7.4%; *P* = 0.041—for vessels). The RMF (by 20.5%; *P* < 0.001) and blood vessel (by 35.2%; *P* < 0.001) amount in the CH+PRP group was higher than that in the CH group.

**Figure 1:**
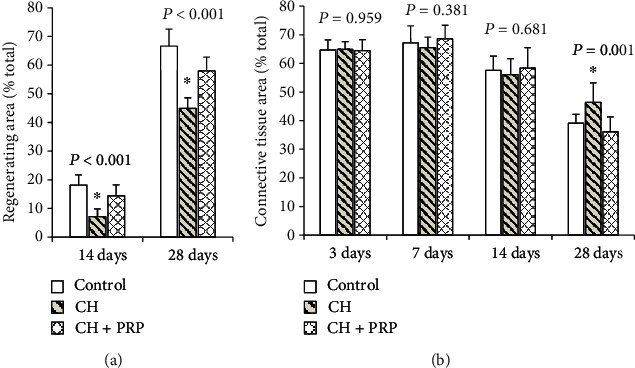
(a) Percentage regenerating area of total muscle fiber cross-sectional area on the 14^th^ and 28^th^ day after injury. (b) Percentage of connective tissue area of total muscle organ cross-sectional area on the 3^rd^, 7^th^, 14^th^, and 28^th^ day after injury. ^∗^Significant difference by ANOVA.

The morphometric analysis also included calculations on the area of regeneration and connective tissue (Figure 1). The regeneration area in rats with CH was significantly smaller compared to that in control animals (by 60.8%; *P* < 0.001—on the 14th day; by 32.6%: *P* < 0.001—on the 28th day) and the CH+PRP group (by 50.7%; *P* < 0.001—on the 14th day; by 22.6%; *P* < 0.001—on the 28th day). A significant difference in connective tissue area was revealed only on the 28th day after muscle lesion. Thus, the connective tissue area in the CH group was larger than that in control rats (by 15.6%; *P* = 0.014) and CH+PRP animals (by 22.1%; *P* = 0.001).

**Figure 2:**
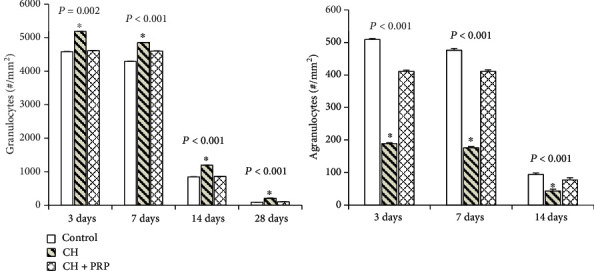
Amount of white blood cells (agranulocytes and granulocytes) in the skeletal muscle on the 3^rd^, 7^th^, 14^th^, and 28^th^ day after the mechanical injury. Data are presented as means ± SD. ^∗^Significant difference by ANOVA.

One of the key components of successful skeletal muscle regeneration is the quality and proper sequence of inflammation development. Thence, the granulocyte and agranulocyte numbers in regenerating muscle were calculated (Figure 2). During the whole period of muscle recovery, the granulocyte amount was found to be significantly higher in rats with CH compared to control and CH+PRP group (*P* < 0.05). In contrast, the agranulocyte content in CH rats was lower than that in control animals and CH+PRP rats (*P* < 0.05).

**Figure 3:**
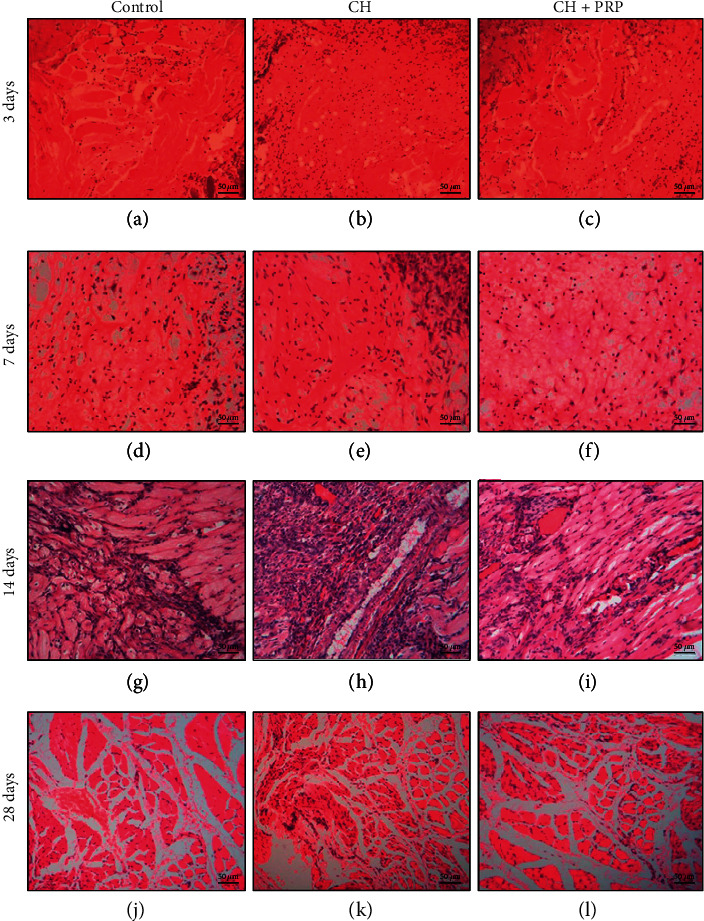
Histology of skeletal muscle regeneration in control (a, d, g, j), chronic hyperglycemia (b, e, h, k), and chronic hyperglycemia+platelet-rich plasma (c, f, i, l) groups on the 3^rd^, 7^th^, 14^th^, and 28^th^ day after mechanical injury. Explanations are in the text. Staining—hematoxylin and eosin. Scale bar—50 *μ*m.

The results of histological analysis in all studied groups are shown in Figure 3. The pronounced muscle fiber necrosis, tissue swelling, blood vessel plethora, and leukocyte infiltration were observed in control rats on the 3rd day after injury (Figure 3(a)). On the 7th day, the large number of fibroblasts and massive collagen fiber formation were detected in the control group (Figure 3(d)). Tissue swelling and leukocyte infiltration were also maintained. On the 14th day, the new muscle fiber formation and significant angiogenesis activation were observed. The slight edema and leukocyte infiltration were also detected. On the 28th day, the histological picture of skeletal muscle recovery in control animals was characterized by fibromuscle regenerate development. There was a massive extracellular matrix formation around CNFs. A significant number of vessel was also observed (Figures 3(g) and 3(j)).

The pattern of muscle regeneration in rats with CH on the 3rd and 7th day after the muscle injury was mostly similar to control animals. However, relatively small vessel number, lipid accumulation, more pronounced inflammation, and connective tissue development were observed (Figures 3(b) and 3(e)). On the 14th day, tissue swelling and significant leukocyte infiltration in CH rats were detected. Necrosis sites together with small newly formed fiber number were observed (Figure 3(h)). On the 28th day, there were massive fibrosis and a relatively small number of newly formed vessels in rats with CH. Muscle fibers had a relatively small area. At the same time, inflammatory infiltration persisted (Figure 3(k)).

The histological picture of muscle regeneration in CH+PRP animals on the 3rd day after lesion mostly corresponded to the CH group. However, on the 7th day, edema and leukocyte infiltration were less pronounced and angiogenesis activity was more noticeable in CH+PRP rats compared to animals with CH (Figures 3(c) and 3(f)). On the 14th day, the massive new muscle fiber formation, blood vessel plethora, and inflammation were detected (Figure 3(i)). On the 28th day, the muscle regeneration process in CH+PRP animals was characterized by the large number of new vessels and by massive fibrosis around the CNFs (Figure 3(l)).

A summary of the qualitative assessment of posttraumatic skeletal muscle regeneration in rats of comparison groups is presented in Table 3.

## 4. Discussion

The structural features of skeletal muscle regeneration in rats with CH, as well as morphological analysis of PRP effect on the striated muscle recovery in animals with CH, were investigated. Mechanical injury by transverse linear incision was used for the experiment. The process of muscle regeneration after the incision had several differences compared to regeneration after injury by chemical agents [10, 26], temperature [26], contusion [11], or straining [14]. Necrosis and inflammation prolongation, as well as massive connective tissue development, were observed. Eventually, the regeneration process has culminated in the formation of fibromuscle regenerate. Herewith, after cardiotoxin [26, 27] or straining [14] injury, complete skeletal muscle regeneration practically repeats the morphological picture of this organ before the trauma.

Histomorphometric analysis of skeletal muscle regeneration in rats with CH revealed several salient features. Thus, edema and leukocyte infiltration were observed even 28 days after injury. Herewith, granulocyte predominance and reduced agranulocyte numbers were observed. Similar results were obtained by Krause et al., which showed the decreased macrophage number within regenerating muscles of rats with type 1 DM [9]. Nguyen et al. also have revealed reduced macrophage amount inside skeletal muscle regenerates of rats with a genetic model of type 2 DM [10]. Recent studies have shown that macrophages are the prerequisite for successful skeletal muscle regeneration [28]. Macrophages act as an important regulatory factor in the process of muscle-specific cambial cell activation [29, 30]. Xiao et al. [31] have shown that macrophage depletion leads to excessive connective tissue development and size reduction of newly formed muscle fibers during skeletal muscle regeneration.

Skeletal muscle regeneration in rats with CH was characterized by significantly reduced new vessel formation, which was also found in animals with a genetic model of type 2 DM [10]. Today, there is no single explanation of angiogenesis impairment within skeletal muscle under CH condition [32–34]. But it is likely that restricted blood supply due to disrupted neoangiogenesis is an important factor of incomplete posttraumatic muscle regeneration.

CH inhibits the MyoD and myogenin expression [5] and leads to MSC reduction in the skeletal muscle [6, 7]. Our results revealed the decrease in amount and total area of newly formed fibers within regenerating striated muscle of rats with CH. Jeong et al. also have shown a significant decrease in CNF number during posttraumatic muscle regeneration in rats with streptozotocin-induced diabetes [8]. In addition, skeletal muscle regeneration in rats with genetic DM models was also associated with a significant decrease in MSC activity and number [9, 10].

Skeletal muscle recovery in rats with CH was characterized also by incompleteness, fat inclusion accumulation, and massive collagen fiber formation, which largely corresponds to results obtained in other similar studies [8–10].

Thus, our results revealed the histopathological signs of CH negative effect on muscle regeneration in rats with a mechanical muscle injury. Currently, some molecular mechanisms of CH influence on skeletal muscle repair are described. The experimental DM induces overactivation of myostatin/TGF-*β* receptor signaling, which in turn inhibits the MSC activation, causing poor muscle regeneration [8]. It was also demonstrated that diabetes results in MSC content and functionality decline due to hyperactivation of the Notch signaling pathway [6]. It is not yet known, whether CH, insulin signaling disruption, or both are the reason for MSC regenerative response impairment. It was also shown that several proinflammatory factors (e.g., interleukin-6 and tumor necrosis factor-*α*) are elevated in diabetic patients [35]. This may be a result of advanced glycation end product overformation [36]. Moreover, in vitro experiments revealed that the hyperglycemic environment induces adipogenic differentiation of muscle-derived stem cells [37]. It is assumed that reactive oxygen species and downstream effector kinases, such as PKC-*β*, play the main role in this process. All the data, gathered above, can serve as an attempt to substantiate the reduction of new muscle fiber amount, inflammation deregulation, and adipocyte presence in regenerating muscles of rats with streptozotocin-induced CH.

A recent review by Setayesh et al. [38] showed that PRP promotes skeletal muscle regeneration through growth factors secreted from activated platelets. Aydin et al. demonstrated that PRP could improve the histopathological grades in wound healing suppressed by corticosteroid [39]. In addition, a few meta-analyses have shown that topical PRP application for diabetic ulcer treatment accelerates wound healing and significantly reduces the number of complications [22–24]. Given the abovementioned, we have decided to investigate the PRP effect on skeletal muscle regeneration in rats with CH.

The results of the histological analysis showed the difference in severity (leukocyte infiltration, edema presence) and quality (granulocyte and agranulocyte number) of inflammation between the CH+PRP group and CH rats, wherein the results in the CH+PRP group were almost the same as in the control. Tsai et al. also demonstrated the reduced amount of CD68-positive and apoptotic cells in the injured skeletal muscle treated with PRP [16]. However, Gigante et al. [12] revealed no difference in the inflammation manifestation during skeletal muscle regeneration between PRP-injected rats and rats without PRP using.

The PRP administration into the skeletal muscle of rats with CH also resulted in neoangiogenesis activation. The vessel number in the regenerating muscle of CH+PRP rats was almost the same as in the control group. The neoangiogenesis intensification during muscular recovery due to PRP using was also revealed by Gigante et al. [12]. The results of our study also showed that PRP contributes to increase of CNF number and total regeneration area in the skeletal muscle of rats with CH. The increase in the number of newly formed fibers during muscle recovery due to PRP injection has also been identified in several studies on animals with different types of mechanical muscle injury [13–15]. These studies also reported collagen area and fibrosis degree reduction in striated muscle regenerates due to PRP administration. Our results also showed that the connective tissue area in CH+PRP rats was smaller compared to that in CH rats.

This is the first report about PRP structural effect on skeletal muscle regeneration in rats with experimental CH. The obtained results revealed that PRP could enhance striated muscle recovery under CH conditions. However, our research had a few important limitations, which have to be taken into consideration. Firstly, the concentration of growth factors and cytokines was not evaluated in the prepared PRP. Secondly, the immunohistochemistry and confocal microscopy were not used. These would have made the evaluation of the neoangiogenesis nature, as well as the cellular composition of regenerating muscles, much more accurate. Thirdly, no molecular-genetic techniques, such as RT-PCR, have been applied to evaluate the CH and PRP effect on specific transcription factor expression. Finally, no analysis of dynamic and strength indices of regenerating skeletal muscle were performed, which made it impossible to assess functional recovery in the comparison groups.

## 5. Conclusion

Thus, the streptozotocin-induced CH has a negative impact on posttraumatic striated muscle regeneration, contributing to massive connective tissue development instead of new muscle fiber formation. The autologous PRP injection promotes the skeletal muscle recovery process in rats with CH, shifting it away from fibrosis toward the complete muscular organ formation.

## Figures and Tables

**Table 1:** The blood biochemical test of control and experimental rats.

Parameter	Control (*n* = 10)	CH (*n* = 10)	*P*
Fasting glucose (mmol/l)	4.97 ± 0.73	14.76 ± 1.87	<0.001
Total cholesterol (mmol/l)	1.89 ± 0.21	3.26 ± 0.36	<0.001
Triglycerides (mmol/l)	0.54 ± 0.11	1.03 ± 0.16	<0.001
LDL (mmol/l)	0.59 ± 0.08	0.93 ± 0.12	<0.001
HDL (mmol/l)	1.92 ± 0.20	1.48 ± 0.21	0.004
Insulin (*μ*MU/ml)	16.01 ± 1.81	12.35 ± 1.77	0.005
С-peptide (ng/ml)	3.47 ± 0.79	3.96 ± 0.64	0.267

LDL: low-density lipoproteins; HDL: high-density lipoproteins; CH: experimental chronic hyperglycemia. Data are presented as means ± SD.

**Table 2:** The number of muscle fibers and vessels in the injured skeletal muscle.

Parameter	Group	3 days	7 days	14 days	28 days
Damaged fibers (no./mm^2^)	Control	210.4 ± 15.9	202.7 ± 23.7	197.2 ± 12.9	47.4 ± 3.5
	CH	227.4 ± 14.6	225.4 ± 14.1	283.3 ± 18.9	150.1 ± 14.1
	CH+PRP	212.8 ± 12.9	193.8 ± 21.7	206.0 ± 32.7	102.6 ± 4.6
	P	0.030	0.005	<0.001	<0.001
Regenerating fibers (no./mm^2^)	Control	—	22.5 ± 2.8	211.5 ± 14.1	512.3 ± 38.8
	CH	—	5.9 ± 0.3	92.9 ± 6.2	375.0 ± 35.2
	CH+PRP	—	15.7 ± 1.8	170.2 ± 26.9	471.9 ± 21.2
	P	—	<0.001	<0.001	<0.001
Vessels, (no./field)	Control	10.7 ± 0.8	11.2 ± 1.5	19.2 ± 1.4	27.0 ± 2.1
	CH	7.5 ± 0.5	7.3 ± 0.7	11.6 ± 0.8	16.2 ± 1.5
	CH+PRP	9.8 ± 0.6	9.4 ± 1.1	16.3 ± 2.6	25.0 ± 1.3
	*P*	<0.001	<0.001	<0.001	<0.001

Data are presented as means ± SD. CH: chronic hyperglycemia; PRP: platelet-rich plasma; *P*: possibility by Fisher *F*-criterion. Results of Bonferroni post hoc test are described in the text.

**Table 3:** Qualitative assessment of skeletal muscle regeneration.

	3 days			7 days			14 days			28 days		
	Ctrl	CH	CH+PRP	Ctrl	CH	CH+PRP	Ctrl	CH	CH+PRP	Ctrl	CH	CH+PRP
Necrosis	+++	+++	+++	++	+++	++	–	+	–	–	–	–
Connective tissue edema	++	+++	+++	+	+++	+	+	++	+	–	+	–
Inflammatory infiltration	+++	+++	+++	+	+++	++	+	++	+	–	+	–
Vascularization	++	+	++	++	+	++	+++	++	++	+++	++	+++
Fibrosis	+	+	+	++	++	++	++	++	++	++	+++	++
CNFs	–	–	–	+	–	+	++	+	++	+++	++	+++

Ctrl: control group; CH: rats with chronic hyperglycemia; CH+PRP: rats with chronic hyperglycemia+platelet-rich plasma injection; CNFs: centrally nucleated fibers. The results are expressed as a percentage of the highest value among all groups: (–) rare or not detected; (+) between 10% and 30%; (++) between 30% and 60%; (+++) over 60%.

## Data Availability

The data used to support the findings of this study are available from the corresponding author upon request.
